# Epidemiology, pathogenesis, and management of takotsubo syndrome

**DOI:** 10.1007/s10286-017-0465-z

**Published:** 2017-09-15

**Authors:** Shams Y-Hassan, Per Tornvall

**Affiliations:** 1 Department of Cardiology, Karolinska Institutet at Karolinska University Hospital, Solna, Sweden; 2 Department of Clinical Science and Education Södersjukhuset, Karolinska Institutet, Sjukhusbacken 10, 118 83 Stockholm, Sweden

**Keywords:** Takotsubo, Broken heart syndrome, Acute coronary syndrome, Neurogenic stunned myocardium, Left ventricle ballooning

## Abstract

Takotsubo syndrome is a recently recognized acute cardiac disease entity with a clinical presentation resembling that of an acute coronary syndrome. The typical takotsubo syndrome patient has a unique circumferential left (bi-) ventricular contraction abnormality profile that extends beyond a coronary artery supply territory and appears to follow the anatomical cardiac sympathetic innervation. The syndrome predominantly affects postmenopausal women and is often preceded by emotional or physical stress. Patients with predisposing factors such as malignancy and other chronic comorbidities are more prone to suffer from takotsubo syndrome. The pathogenesis of takotsubo syndrome is elusive. Several pathophysiological mechanisms involving myocardial ischemia (multivessel coronary artery spasm, microvascular dysfunction, aborted myocardial infarction), left ventricular outlet tract obstruction, blood-borne catecholamine myocardial toxicity, epinephrine-induced switch in signal trafficking, and autonomic nervous system dysfunction have been proposed. The syndrome is usually reversible; nevertheless, during the acute stage, a substantial number of patients develop severe complications such as arrhythmias, heart failure including pulmonary edema and cardiogenic shock, thromboembolism, cardiac arrest, and rupture. Treatment of precipitating factors, predisposing diseases, and complications is fundamental during the acute stage of the disease. The epidemiology, pathogenesis, and management of takotsubo syndrome are reviewed in this paper.

## Introduction

**Fig. 1:**
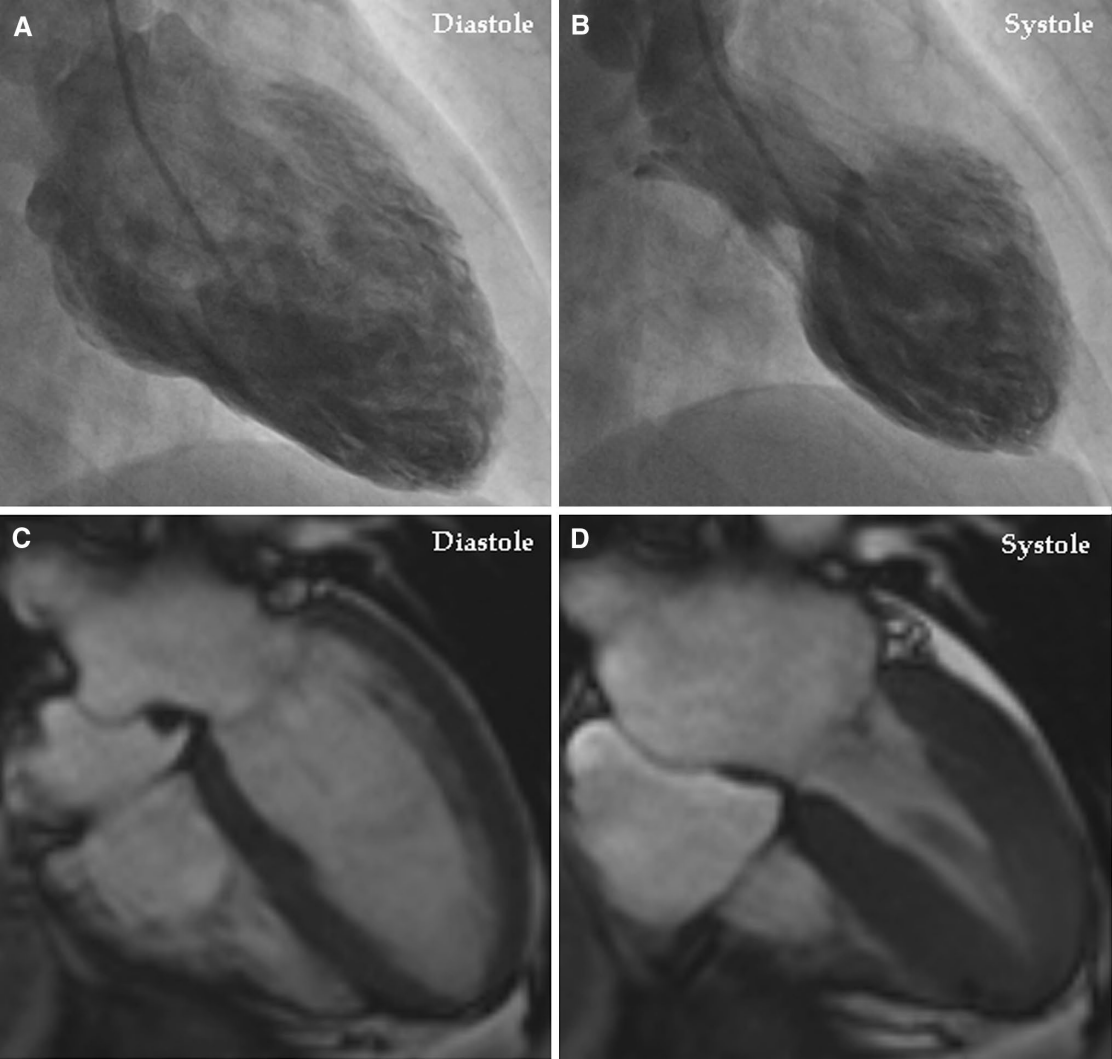
Left ventriculography during the acute stage of takotsubo syndrome shows typical midapical ballooning during systole (**a** diastole; **b** systole). Cardiac magnetic resonance imaging 4 days after left ventriculography shows complete normalization of the left ventricular function (**c** diastole; **d** systole)

**Fig. 2:**
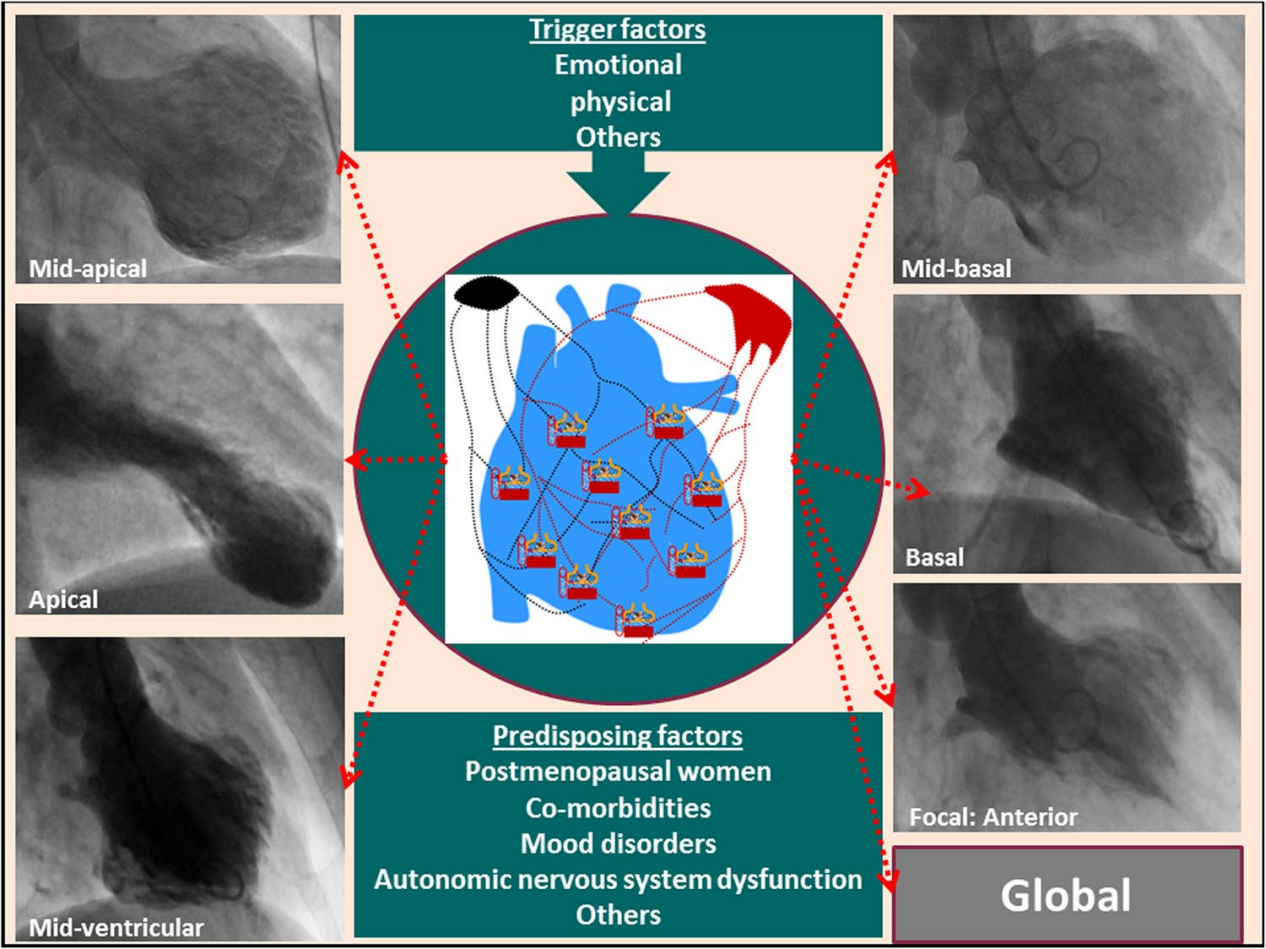
An emotional or a physical trigger in predisposed individuals may result in diverse left ventricular contraction patterns (midapical, apical, midventricular, midbasal, basal, focal, and global). The figure is modified from Y-Hassan S and De Palma R [1] with copyright permission

Takotsubo syndrome (TS), also known as broken heart syndrome or neurogenic stunned myocardium, is a recently recognized acute cardiac disease entity [1]. The term takotsubo (tako = octopus, tsubo = a pot) was introduced by Sato and Dote in 1990 and 1991 to describe the left ventricular silhouette during systole in five patients presenting with clinical features of myocardial infarction but without obstructive coronary artery disease [2, 3]. The syndrome has a clinical and electrocardiographic presentation resembling that of an acute coronary syndrome (ACS). The main characteristic feature of TS is the regional left ventricular wall motion abnormality (LVWMA) with a peculiar circumferential pattern resulting in a conspicuous ballooning of the left ventricle during systole (Fig. 1a, b). The LVWMA extends beyond the coronary artery supply regions and is reversible with almost complete resolution of ventricular dysfunction in hours to weeks (Fig. 1c, d). The LVWMA may be localized to the apical, midapical, midventricular, midbasal, or basal (Fig. 2) segments of the left ventricle [1]. A focal or global left ventricular contractile abnormality has also been reported [4, 5]. The right ventricle is involved in about one third of the TS patients [6]. The syndrome is preceded by a trigger factor in about 70% of patients [4]. Emotional triggers such as death of a close relative or acute grief may trigger the syndrome and hence the term broken heart syndrome [7]. Innumerable physical triggers, extending from serious diseases such as intracranial hemorrhages and sepsis to physiological processes such as sexual intercourse and pregnancy, may also trigger the syndrome [8]. Recently, the Heart Failure Association of the European Society of Cardiology in a position statement from the task force on TS introduced the terms primary and secondary TS [9]. In primary TS, acute cardiac symptoms are the primary reason for seeking acute medical care. In secondary TS, the syndrome occurs in patients already hospitalized for a medical or surgical condition. The authors also introduced new diagnostic criteria for TS. Compared to the Mayo Clinic diagnostic criteria for TS [10], which has been adopted for many years, pheochromocytoma is no longer an exclusion criterion for TS.

## Epidemiology

**Fig. 3:**
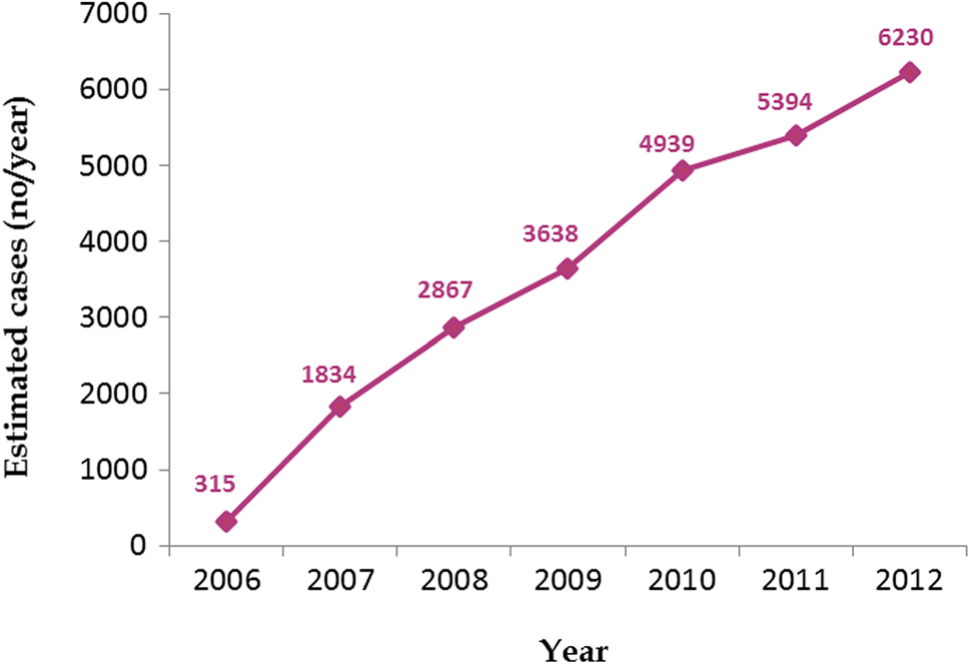
Trends in reported incidence of takotsubo syndrome from 2006 to 2012. Modified from a table by Minhas AS, Hughey AB, Kolias TJ [13]

Prior to the introduction of the term takotsubo, the syndrome existed under different diagnoses. Since the introduction of the Japanese term takotsubo in 1990 [2], the syndrome has been increasingly recognized in almost all countries of the six continents of the world. TS has been reported in a variety of races but according to some reports, it is uncommon in Hispanics and African Americans [11]. The prevalence of TS has been reported to be approximately 2% (up to 10% if only women are considered) of all patients presenting with clinical manifestation of ACS [12]. The prevalence is underestimated and the main reason for this is unawareness of the disease. However, with increasing awareness and more widespread access to early invasive coronary angiography, TS is now recognized more frequently. Minhas et al. [13] reported on a significant increase in the incidence of TS from 2006 to 2012. In that study, the incidence of TS increased almost 20 times during the time-period (Fig. 3). Similarly, a study by Murugiah et al. [14] showed that hospitalization rates for TS are increasing. In that study, the incidence of primary TS increased from 2.3 hospitalizations per 100,000 person-years in 2007 to 7.1 in 2012. The corresponding incidence for “secondary TS” increased from 3.4 hospitalizations per 100,000 person-years in 2007 to 10.3 in 2012. Studies on TS from different parts of the world have reported that 85–90% of the patients with TS are women, aged 65–70 years [8, 9]. Of 1750 patients with TS, recently reported by Templin et al. [4], 89.8% were women with a mean age of 67 years. However, the syndrome has been reported in both genders and in all age groups and even in children [4, 8, 9]. The prevalence of affected men with TS is increased when the syndrome is triggered by physical stress as severe critical medical illnesses [15, 16]. The syndrome may recur and the recurrence rate of TS has ranged from 0 to 22% [4, 12]. In patients with pheochromocytoma-triggered TS, a recurrence rate of 17.7% has been reported [15], which is most probably due to undiagnosed pheochromocytoma where the trigger remains. Singh et al. [17] reported that the annual recurrence rate was 1.5% and that the cumulative incidence of recurrence was 1.2% at 6 months and 5% at 6 years.

## Pathophysiology

Several pathophysiological mechanisms for the development of TS have been proposed. The pathophysiology of TS is complex and may be multifactorial. The main proposed mechanisms are myocardial ischemia, left ventricular outlet tract obstruction (LVOTO), blood-borne catecholamine myocardial toxicity, epinephrine-induced switch in signal trafficking, and autonomics nervous system dysfunction with sympathetic nervous system hyperactivation including local cardiac sympathetic disruption and norepinephrine seethe and spillover.

### Myocardial ischemia

#### Multivessel coronary artery spasm

When Sato and Dote introduced the term takotsubo in 1990 [3, 18] to describe the left ventricular silhouette during systole, they deemed the left ventricular dysfunction as myocardial stunning due to simultaneous multivessel coronary vasospasm. This was based on the fact that the investigators demonstrated spontaneous multivessel coronary vasospasm at coronary angiography in two patients and observed coronary spasm in two further patients after ergonovine administration. In subsequent series, the prevalence of coronary vasospasm has been variable. Spontaneous coronary vasospasm was reported in 5–10%, which implies that the majority of the patients had no spontaneous coronary vasospasm [12]. Provocation-induced coronary vasospasm has been reported in a limited number of patients in the acute phase of TS. In a review of nine studies, provocation testing could induce coronary vasospasm in only 34 of 123 patients (28%) [12]. In addition to the absence of spontaneous or provocation-induced vasospasm in most cases, LVWMAs with an apical-sparing pattern argue against the multivessel coronary spasm hypothesis in causing TS. Furthermore, dobutamine, which is a vasodilator with minimum vasospastic effects, has been reported to induce TS [19]. Likewise, epinephrine which also has a dominant coronary vasodilatory effect, has been reported to induce TS [19, 20].

#### Microvascular dysfunction

Microvascular dysfunction has been advocated by several investigators as a possible pathophysiological mechanism of LVWMA in TS [21]. Coronary angiography reveals normal coronary arteries in the majority of patients with TS [4]. One important limitation of coronary angiography is its inability to visualize the coronary microcirculation. Surrogate noninvasive and invasive techniques have been used to study the microcirculation and the findings may support involvement of the coronary microcirculation in the pathogenesis of TS. Studies using semiquantitative invasive techniques as thrombolysis in myocardial infarction (TIMI) frame count, corrected TIMI frame count (CTFC), TIMI myocardial perfusion grade (TMPG), and coronary flow reserve (CFR) have shown conflicting results. By assessing TIMI frame count, Fazio et al. [22] reported slow coronary flow in 23 of 24 patients with TS during the acute stage. However, the authors observed some findings which argue against the role of microvascular dysfunction, as only nine patients had slow flow in all three epicardial coronary arteries. In another study, Sharkey et al. [23] did not find significant differences in TIMI frame count measurements in 59 patients with TS compared with controls.

Noninvasive methods such as Doppler transthoracic echocardiography (TTE) and myocardial contrast echocardiography (MCE) can provide analysis of both LVWMA and coronary flow [21]. Studies using these noninvasive techniques have shown lower myocardial blood flow in dysfunctional left ventricular segments compared with left ventricular segments showing normal wall motion in the acute phase in patients with TS [21, 24]. TTE-CFR has also been used to study coronary flow in the acute phase of the disease with similar results [21]. There are a few studies performed after the acute stage of TS. In a study of 22 TS patients 1 year after the acute TS event, Collste et al. [25] could not show decreased TTE-CFR in the left anterior descending artery when compared with healthy controls during high-dose dobutamine administration, indicating that the microvascular dysfunction seen during the acute stage may be a secondary phenomenon.

#### Aborted myocardial infarction caused by a transient thrombosis in a long wrap-around left anterior descending artery (LAD)

In a study of five patients with TS, Ibanez et al. [26] reported disrupted atherosclerotic plaques in a long wrap-around LAD when studied by intravascular ultrasound (IVUS). In contrast, other investigators [27, 28] could not confirm disrupted atherosclerotic plaques in TS patients with IVUS performed during the acute stage. The apical-sparing patterns in midventricular and basal types of TS argue against aborted myocardial infarction in a long wrap-around LAD as a cause of TS. The apical-sparing patterns also argue against myocardial bridging with systolic compression of LAD as a cause [29–31]. Furthermore, in 73% of patients with TS, the course of LAD does not fulfil the criteria of long wrap-around LAD [32]. Long-lasting (hours or even days) ST segment elevation in patients with TS also challenges the hypothesis of aborted myocardial infarction, in which rapid resolution of ST elevation after reperfusion is an important feature [33, 34].

Another feature in TS, which could not be explained by the proposed ischemia hypothesis, is the cardiac myocyte histopathology. Contraction band necrosis is a characteristic histopathologic feature in TS that is distinct from coagulation necrosis, the fundamental histopathological sign in myocardial infarction [35–37]. Worth mentioning is the fact that TS and chronic obstructive coronary artery disease may coexist [4]. Furthermore, ACS caused by acute thrombotic coronary occlusion or spontaneous coronary artery dissection with associated severe chest pain is a major stress factor and, as any other physical stress factor, may trigger TS [38–40].

### Left ventricular outlet tract obstruction (LVOTO)

LVOTO has been observed in patients with TS. This may cause hypotension and may be one of the causes of cardiogenic shock in TS. El Mahmoud et al. [41] demonstrated a relatively high prevalence of LVOTO (25%) in a study of 32 TS patients. They suggested that patients with a localized sigmoid septum and a small left ventricle may be predisposed to severe midcavity obstruction during periods of excessive sympathetic stimulation. Theoretically, this LVOTO could result in apical subendocardial ischemia with resulting ballooning due to a large pressure gradient between the apex and base of the left ventricle. However, the majority of patients with TS do not have LVOTO [41]. Furthermore, LVOTO does not provide a reasonable explanation for the apical-sparing and basal patterns of TS, which is found in substantial numbers of patients [15, 20, 29, 30, 42, 43]. Furthermore, the right ventricular involvement seen in almost one third of the patients with TS cannot be explained by LVOTO [6, 44]. The LVOTO observed in some patients with TS is most likely a complication rather than an underlying cause of the LVWMA.

### Blood-borne catecholamine myocardial toxicity

Blood-borne catecholamine myocardial toxicity has been suggested as one of the pathophysiological mechanisms of TS. This is based on the history of emotional stress preceding the disease onset in many patients with TS [8]; the report of extremely high plasma catecholamine levels in TS patients in one study [45]; the occurrence of TS in patients with pheochromocytoma [15]; and the induction of TS by therapeutic and accidental administration of epinephrine, norepinephrine, and other catecholamine inotropics [20, 46]. However, this hypothesis is challenged by other findings in patients with TS. Clinically, a considerable number of patients with TS lack a history of antecedent emotional or physical triggering stress factor [8, 47]. Extremely high plasma catecholamine levels have not been reproduced in other studies [48–50]. Furthermore, the circumferential pattern of LVWMA with sparing of basal or apical parts of the left ventricle (Fig. 2) indicates that blood-borne catecholamines (if increased as in pheochromocytoma) are not primarily causing TS but rather act a trigger factor for the cardiac sympathetic system to cause TS [19].

### Plasma cortisol and TS

On one hand, TS triggered by adrenal adenocarcinoma secreting high cortisol levels has been reported [51]. On the other hand, TS triggered by secondary adrenal insufficiency in ACTH isolated deficiency has also been reported [52]. Evidence for a causal relation between plasma cortisol and TS is lacking. Madhavan et al. [48] reported no differences between plasma cortisol levels in patients with TS and patients with ST elevation myocardial infarction. The 24-h urine free cortisol levels were normal in all patients with TS. These results were confirmed by another study reported by Kastaun et al. [53]. Smeijers et al. investigated the hypothalamic-pituitary-adrenal (HPA) axis (adrenocorticotropic hormones and cortisol) in patients with a past history of TS. They found the expected HPA responses to mental stress with no difference between TS and healthy controls [54].

### Epinephrine-induced switch in signal trafficking

Lyon et al. [55] in 2008 hypothesized that high levels of circulating epinephrine trigger a switch in the intracellular signal trafficking from Gs (stimulatory) to Gi (inhibitory) protein signaling through the B2 adrenoreceptor (B2AR). They suggested that this change in signaling was negatively inotropic and that the effect was greatest at the apical myocardium, explaining the apical ballooning seen in TS. In a rat model of TS, Paur et al. [56] reported that an injection of a high-concentration bolus dose of epinephrine triggered left ventricular apical ballooning. They suggested biased agonism of epinephrine for Gs at low concentrations compared with Gi at high concentrations explained the LVWMA observed in TS. However, there are clinical findings in TS patients that challenge this suggested pathophysiological mechanism. Paur et al. based their suggestion on the presumed fact that in patients with TS, there will be an extreme surge of epinephrine in response to the triggering stressor or coexisting medical condition. However, extremely high plasma concentrations of catecholamines have only been seen in one study [45], whereas other studies have shown normal or near normal plasma catecholamine levels [48–50]. Paur et al. reported in their study only apical ballooning after a high-concentration bolus epinephrine injection. In contrast, Redfors et al. [57] induced mainly the basal type of TS during injection of epinephrine in rats. Furthermore, other investigators [15, 20, 42, 43] reported on apical-sparing patterns of TS in almost 50% of patients when triggered by epinephrine administered either therapeutically or accidentally or TS triggered by pheochromocytoma. Consequently, substantial evidence argues against a direct causal effect of epinephrine in TS; nevertheless, epinephrine may act as a trigger factor for TS.

### Hyperactivation of the sympathetic nervous system in the pathogenesis of TS

**Fig. 4:**
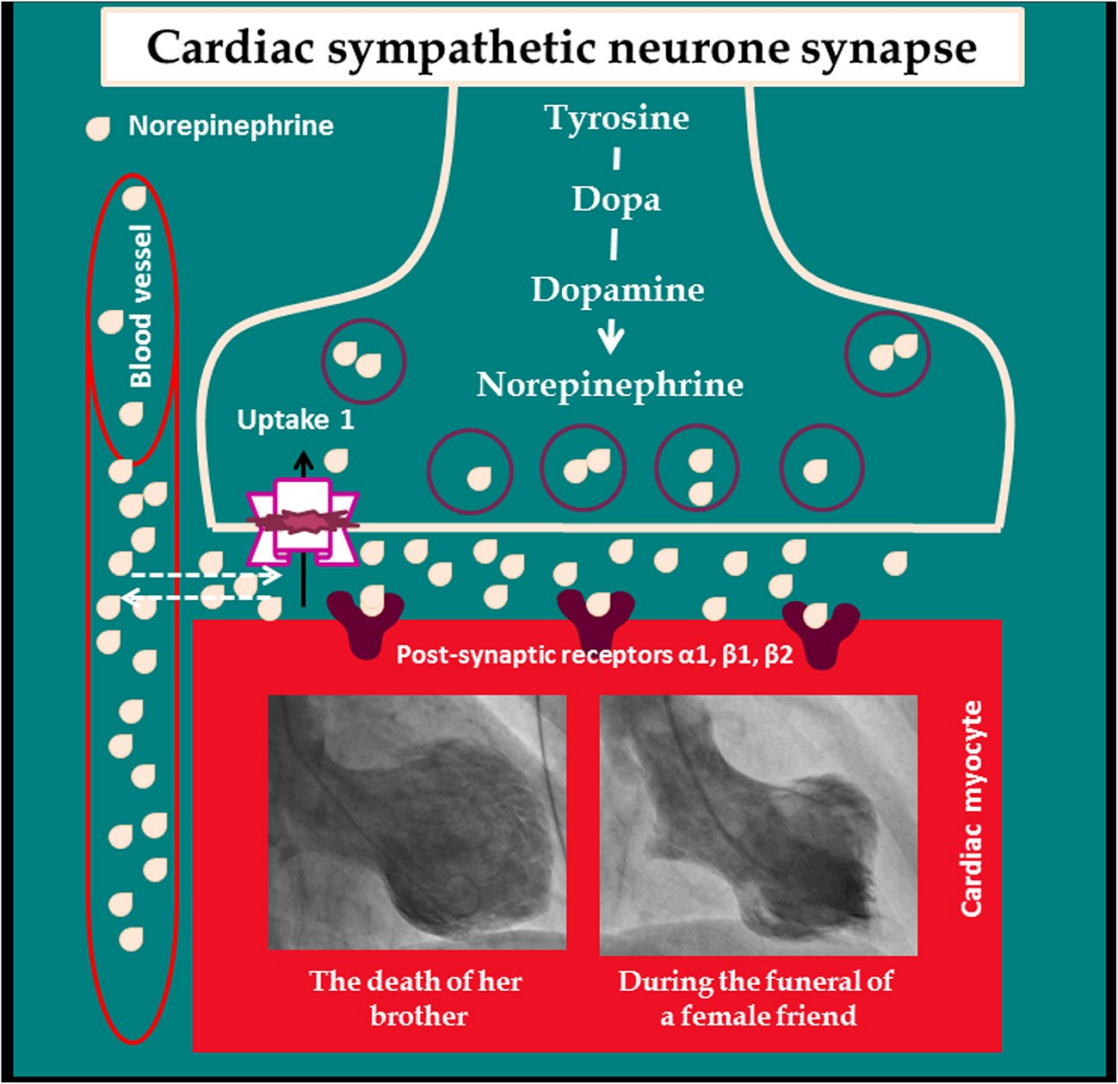
Illustration of cardiac sympathetic hyperactivation disruption at the cardiac sympathetic neurone synapse. Under normal physiologic conditions, when a cardiac sympathetic nerve is stimulated, norepinephrine stored in granules in the presynaptic nerve terminals is released into the synaptic cleft. Norepinephrine stimulates postsynaptic alpha and beta adrenergic receptors on cardiac myocytes, activating downstream effector pathways. In takotsubo syndrome, local cardiac sympathetic overactivation-disruption results in excess and spillover of norepinephrine, causing regional myocardial stunning, as depicted by left ventriculography of two cases showing midapical and midventricular TS. Norepinephrine excess may also inhibit the uptake-1 region, leading to a decrease of norepinephrine reuptake and increase wash-out of norepinephrine into the circulation. The figure is modified from Y-Hassan S. and De Palma R. [1] with copyright permission

Investigators [1, 58, 59] have provided substantial evidence for involvement of the sympathetic nervous system, including hyperactivation and disruption of local cardiac sympathetic nerve terminals and norepinephrine seethe and spillover, in the pathophysiology of TS (Fig. 4). The occurrence of the syndrome after emotional triggering events, the myocardial stunning with a peculiar circumferential pattern, which may follow the local cardiac sympathetic nerve distribution, the histopathological findings of contraction band necrosis, and the evidence for catecholamine myocarditis with myocardial edema are among supportive data for the involvement of the local cardiac sympathetic nervous system in the pathophysiology of TS [60] discussed below.

#### Evidence for sympathetic nervous system hyperactivation-disruption in TS

##### Emotional stressors

The deep anguish that arises from bereavement and induces TS in an individual argues for an excessive sympathetic stimulation of the myocardium, likely mediated via the brain. Anecdotal histories related to death induced by emotions have been described for hundreds of years [2]. The term “broken heart” was used under circumstances in which life experiences could become capable of causing cardiac death. In 1967, Rees and Lutkins [61] reported on a significant increase in death rates among bereaved close relatives. After the introduction of the term takotsubo in 1990, case series of emotionally triggered TS were reported [47, 62]. In larger observational studies, TS triggered by emotions comprises more than one fourth (27.7%) of the patients reported with TS [4].

##### Acute brain diseases and TS

Acute intracranial diseases such as intracranial and subarachnoid hemorrhage, thrombotic stroke, epilepsy, and others have been reported to induce TS [63–67]. This strongly suggests the involvement of the sympathetic nervous system in the pathophysiology of TS. This may occur through an increase in intracranial pressure causing hyperactivation of the cardiac sympathetic nervous system [68]. Hammer et al. [69] reported on the rapid appearance and disappearance of the electrocardiographic changes with perturbations of the nervous system. Sharkey et al. [70] reported on episodes of decerebrate posturing in a comatose patient who had sustained a closed head injury with LVWMA. With each episode, transient anterior ST segment elevation appeared followed by resolution of ST elevation and finally T-wave inversion. These findings suggest that these effects were due to neural rather than humoral factors.

##### Medical or surgical sympathetic antagonism and TS

Propranolol, which is a beta-adrenoceptor antagonist, has shown cardioprotective effects in patients with subarachnoid hemorrhage [71]. Necrotic myocardial lesions were present in all patients who received placebo, whereas no necrotic lesions were observed in patients treated with propranolol, suggesting a cardioprotective effect of antagonism of sympathetic activation in the course of subarachnoid hemorrhage. Furthermore, preadmission treatment with beta-blockers in patients with aneurysmal subarachnoid hemorrhage was associated with a decreased incidence of neurogenic stunned myocardium [72]. Other investigators have reported on similar cardioprotective effects of beta-blockers in the course of subarachnoid hemorrhage [73]. ECG changes resembling those observed in the midapical pattern of TS and TS-like LVWMA have been induced in animal studies by brain and stellate ganglion stimulation, and experimental intracranial haemorrhage [60]. These changes could be prevented either by surgical (spinal cord transection or severing) or pharmacological (reserpine, propranolol) sympathectomy [1, 60]. Furthermore, Sharkey et al. [70] could interrupt cycles of anterior ST elevations during episodes of decerbrate posturing in a patient with a closed head injury and LVWMA by administering metoprolol and clonidine.

##### Prevalence of diabetes mellitus in patients with takotsubo syndrome

A low prevalence of diabetes mellitus in TS patients has been reported by several investigators. In a review of 959 studies, Madias [74] reported that the prevalence of diabetes mellitus among patients with TS to be within the range 10.2–17%, which was lower than the 26.9% found by the National Health and Nutritional Examination Survey (NHANES). Tornvall et al. [75] confirmed this finding in a recent register study where diabetes mellitus was significantly less common in TS (6.5%) compared to ACS patients (19.8%). Diabetes mellitus-induced autonomic neuropathy may lead to brain–heart disconnection and thus may have a protective effect against TS in situations with emotional or physical stress.

#### Evidence for local cardiac sympathetic denervation in TS

Signs of cardiac sympathetic denervation assessed by 123I-metaiodobenzylguanidine (^123^I-MIBG) scintigraphy [76] and by ^11^C hydroxyephedrine positron emission tomography (PET) [77] have been demonstrated at LVWMA regions in patients with TS. Madias [78] reviewed 112 TS patients who underwent ^123^I-MIBG. The principle finding from the analysis was decreased regional uptake of ^123^I-MIBG in hypokinetic/akinetic left ventricular segments with increased washout of ^123^I-MIBG.

#### Norepinephrine seethe and spillover from the cardiac sympathetic nerve terminals

Induction of brain death and subarachnoid haemorrhage in animal models and diverse types of intracranial diseases and injuries in humans have been reported to induce TS-like LVWMA [1]. Increased myocardial interstitial, but not plasma, norepinephrine has been demonstrated by Mertes et al. after brain death induction in pigs [79]. Naredi et al. [80] demonstrated increased in total-body norepinephrine concentrations during the acute stage of subarachnoid haemorrhage, suggesting massive sympathetic nervous system activation. Elevated norepinephrine levels in the coronary sinus in five patients with TS demonstrated by Kume et al. [81] suggests increased local myocardial catecholamine release. One further study has shown that sympathectomy, but not adrenalectomy, prevented LVWMA in baboons after subarachnoid haemorrhage, further supporting the role of local catecholamine release in the pathophysiology of the disease [82].

#### Evidence of catecholamine myocarditis in TS

Chemical myocarditis may be one phase in the course of myocardial pathological changes occurring in TS, most probably through local cardiac sympathetic overactivation-disruption and norepinephrine spillover [60, 83, 84]. In a typical TS patient, endomyocardial biopsy revealed myocarditis where catecholamine myocarditis could not be excluded [85]. During recent years, case series of typical TS with biopsy-proven myocarditis or findings consistent with myocarditis on cardiac magnetic resonance (CMR) imaging have been described [84]. In 2012, Neil et al. [86] reported on the extent of myocardial edema detected by CMR imaging, performed in the acute stage and after 3 months, in 32 patients with TS. They found that TS was associated with a slowly resolving global myocardial edema. When changes suggestive of myocarditis are detected by CMR imaging in patients with TS, they are usually constrained to the akinetic/hypokinetic regions of the left ventricle [87]. In a CMR imaging study of a large TS patient group, Eitel et al. [6] demonstrated myocardial edema in 162 of 199 patients (81%) with a distinct transmural, midventricular to apical regional distribution pattern matching the distribution of the LVWMA. Focal or patchy nonischemic late gadolinium enhancement was detected in 22 of 239 patients (9%). In the same study, 110 of 164 (67%) patients who underwent inflammation assessment according to the Lake Louise consensus criteria for CMR diagnosis of myocardial inflammation/myocarditis had evidence of active inflammation. Of those patients, 75% had concomitant pericardial effusion, providing supportive evidence of an inflammatory process in the acute phase of the disease.

#### The distribution pattern of the regional ventricular wall motion abnormality in TS

The LVWMA in TS has a characteristic systematized pattern. It affects the ventricular myocardium circumferentially with a sharp transition between the hypo/akinetic and the normal/hyperkinetic myocardial segments, resulting in a conspicuous left ventricular ballooning during systole [88]. The heart is densely innervated with sympathetic nerves which are distributed on a regional basis. The pattern of LVWMA in TS appears to follow the anatomical sympathetic innervation from the left and right stellate and caudal ganglia [89] (Fig. 2). Worth mentioning is that there are only two anatomical systems which may be involved in the LVWMA on a regional basis: the coronary arterial and the neural systems. Convincing evidence challenges the coronary arterial system as being involved in LVWMA in TS, thus making the neural system an attractive pathophysiological substrate [60]. Hence, the different patterns of TS are possibly seen due to involvement of different branches of the cardiac sympathetic system. To the best of our knowledge, the precise anatomical distribution of the various branches of the cardiac sympathetic nervous system is not known, which is necessary to specify the branch or branches of the cardiac sympathetic nerves involved in TS. Such data may facilitate categorization of the different patterns of TS (apical, midapical, midventricular, midbasal, basal, focal and global) according to the sympathetic branch involved (Fig. 2) in a manner analogous to that of a coronary artery occlusion causing anterior, inferior, lateral, and true posterior myocardial infarction.

## Autonomic nervous system and TS

Heart rate variability (HRV) analysis has been performed in both the acute phase and at follow-up of TS by several investigators [90]. The indices of HRV were markedly depressed within 48 h after hospital admission but normalized at the 3-month follow-up [91]. Bonnemeier et al. [92] reported significant differences in cardiac autonomic modulation and fractal organization of heart rate dynamics between apical and midventricular ballooning patterns of TS. The authors concluded that inhomogeneous efferent bilateral sympathetic coactivation and differences in reflex autonomic regulation may be the underlying pathophysiological mechanisms for apical and midventricular patterns of TS. Vaccaro et al. [93] reported that patients with TS in the subacute phase of the disease exhibited elevated sympathetic nervous system activity associated with a decrease in spontaneous baroreflex control of sympathetic activity. In contrast, Sverrisdottir et al. [94] found that patients with TS had a decrease in muscle sympathetic nerve activity compared with healthy matched controls. However, more than half of the subjects in this later study were studied in the recovery phase up to 6 months after their TS event. Impaired baroreflex control may play a role in the development of TS; even long after the initial episode, women with previous episode of TS have excessive sympathetic responsiveness and reduced parasympathetic modulation of their heart rate [95, 96]. Worsening of chest pain and T-wave inversions on electrocardiogram have been reported in a patient with TS who received atropine because of bradycardia [97]. The withdrawal of the parasympathetic drive may thus exacerbate the sympathetic activity, resulting in worsening of the LVWMA. During follow-up of TS, Collste et al. [98] reported that mental stress performed more than 6 months after the acute event did not induce a significant difference in myocardial function or HRV response between TS and control subjects. However, Lazzeroni et al. [99] reported that compared to healthy subjects, TS patients, investigated more than 1 year after the acute event, showed blunted parasympathetic reactivation after exercise, thereby suggesting that vagal control of heart rate is abnormal in TS.

## Management of TS

### Confirm the diagnosis of TS

TS may be misdiagnosed as ACS because of the similarities in clinical presentation. TS should, therefore, be considered as a differential diagnosis in patients presenting with clinical features of ACS. TS may be triggered by, among others, intracranial hemorrhage or gastrointestinal bleedings and treating such patients with anticoagulation and fibrinolysis may result in life-threatening bleeding complications and death. Cardiogenic shock caused by LVOTO in TS may be misdiagnosed as cardiogenic shock due to extensive myocardial infarction and treated by catecholamine inotropics, which may result in augmentation of LVOTO and worsening of the cardiogenic shock with dismal consequences. Performing urgent coronary angiography, including left ventriculography, is essential to detect TS and to differentiate it from ACS because patients with TS have no coronary culprit lesion to explain the entire LVWMA seen in TS. Furthermore, the association between ACS and TS should not be missed since ACS may trigger TS [100]. In women presenting with ACS without angiographically demonstrable obstructive coronary artery disease, other mechanisms of myocardial infarction with TS as coronary ulceration with peripheral embolization, transient coronary vasospasm, or spontaneous coronary artery dissection should be considered [40, 101, 102]. In patients with severe comorbidities, where invasive coronary angiography may be associated with risks of complications, noninvasive cardiac computed tomography angiography may be considered [40]. Cardiac imaging studies, especially echocardiography, should be performed as early as possible during patient presentation and should be repeated because the LVWMA may resolve in hours to weeks [100] (Fig. 2). Echocardiography is also valuable to identify the distribution of LVWMA, involvement of the right ventricle, and complications of TS as left ventricular thrombus and LVOTO. CMR imaging provides invaluable information on the morphology of TS, involvement of the right ventricle, myocardial edema, and complications of TS as left ventricular thrombus. Furthermore, CMR imaging can differentiate TS from diseases which cause irreversible myocardial damage, such as myocardial infarction and myocarditis [6].

### Treatment of the precipitating and predisposing diseases

**Fig. 5:**
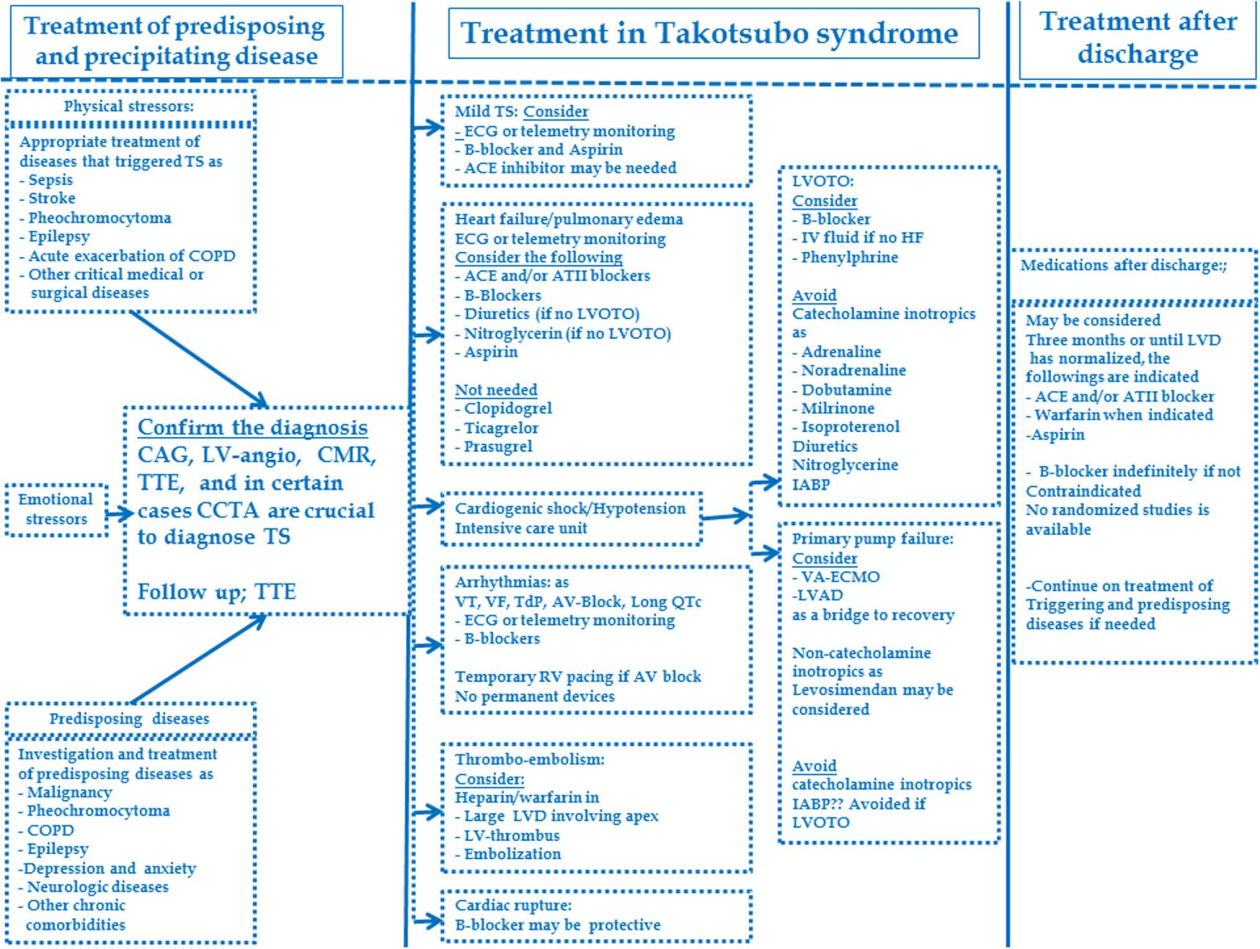
Management of takotsubo syndrome. *ATII* angiotensin II, *CAG* coronary angiography, *COPD* chronic obstructive pulmonary disease, *ECG* electrocardiogram, *IABP* intra-aortic balloon pump, *HF* heart failure, *LV* left ventricular, *LVAD* left ventricular assist device, *LVD* left ventricular dysfunction, *LVOTO* left ventricular outlet tract obstruction, *TdP* Torsades de pointes, *TS* takotsubo syndrome, *TTE* transthoracic echocardiography, *VA-ECMO* venoarterial-extracorporeal membrane oxygenation, *VF* ventricular fibrillation, *VT* ventricular tachycardia

In almost one third of the patients, TS is triggered by physical stress, including serious medical or surgical conditions as sepsis, intracranial diseases, pheochromocytoma, and acute exacerbation of chronic obstructive pulmonary diseases [8, 15, 103]. In a study comprised of 1750 patients [4], 36% of TS patients had physical stress as a trigger. These diseases may contribute significantly to the increase in mortality seen in patients with TS [104]. Furthermore, in undiagnosed pheochromocytoma [75], there is an increased risk of recurrent TS, sometimes with a dramatic clinical presentation including a compromised hemodynamic situation [15, 105]. Proper investigations for predisposing diseases including appropriate treatment in addition to treatment of TS is imperative and may have an impact on improving the prognosis and in preventing TS recurrence (Fig. 5).

### Treatment of TS

#### Treatment during the acute stage

A characteristic feature of TS is the spontaneous resolution of LVWMA in hours to weeks [4, 8, 100] (Fig. 1). Consequently, treatment in the acute stage should be supportive and focus on appropriate treatment of complications [4, 9]. There are neither controlled studies nor guidelines on how to treat TS. In mild cases, in addition to supportive therapy, beta-blockers and aspirin may be considered [9, 106]. In cases complicated by heart failure, conventional treatment with angiotensin-converting enzymes (ACE) and/or angiotensin receptor blockers, beta-blockers, and diuretics is often initiated [4, 107]. However, one study found no association between early beta-blocker use and in-hospital mortality in patients with TS [108]. TS patients should be monitored by telemetry and arrhythmias should be treated accordingly [9]. One serious complication of TS is thromboembolism [109]. In patients with extensive midapical ballooning and documented thrombus in the left ventricle or embolic complications, anticoagulation is recommended for 2–3 months or until resolution of LVWMA and left ventricular thrombus has been confirmed by echocardiography [9, 109]. Beta-blockers, theoretically, may have a protective effect against cardiac rupture, which also is a recognized complication of TS [110].

#### Treatment of cardiogenic shock in TS

Cardiogenic shock is an important and life-threatening complication in TS [4, 8, 15]. The crucial step in treatment is to detect whether the hypotension is caused by LVOTO or primary pump failure [111]. In LVOTO, which may be associated with systolic anterior motion (SAM) of the mitral valve anterior leaflet and mitral regurgitation, catecholamine inotropics such as adrenaline and noradrenaline, dopamine, milrinone, and isoproterenol are contraindicated. Treatment with diuretics, nitroglycerine, and an intraaortic balloon pump should be avoided because of the risk of aggravation of LVOTO. The suggested treatment in LVOTO is intravenous fluid and parenteral beta-blockers, which increases cardiac filling and suppresses the basal hypercontractility, thereby reducing LVOTO [112]. In patients intolerant to intravenous fluid and beta-blockers, phenylphrine may be effective by increasing the afterload and the left ventricular size. In primary pump failure, treatment with venoarterial extracorporeal membrane oxygenation or a left ventricular assist device should be considered as a bridge to recovery [9]. Noncatecholamine inotropics such as levosimendan [113] may also be considered in primary pump failure in TS but catecholamine-based inotropics should be avoided [9].

#### Treatment after discharge

There are no randomized studies on long-term treatment in TS. Observational studies have shown that the use of ACE inhibitors or angiotensin receptor blockers was associated with improved survival up to 1 year after the acute event. Such survival benefits have not been observed with beta-blockers [4]. As mentioned above, in cases of left ventricular thromboembolism, treatment with warfarin should be continued until resolution of the LVWMA and left ventricular thrombus. Appropriate treatment and prophylaxis of the precipitating and predisposing diseases is vital to prevent recurrence of the disease.
